# Evaluation of ergonomic work postures among dental students during restorative procedures using an artificial intelligence–based assessment model

**DOI:** 10.3389/froh.2026.1790719

**Published:** 2026-04-29

**Authors:** Sanjeev B. Khanagar, Aram Alshehri, Farraj Albalawi, Sara Kalagi, Maryam A. Alghilan, Mohammed Awawdeh, Kiran Iyer

**Affiliations:** 1 Preventive Dental Science Department, College of Dentistry, King Saud bin Abdulaziz University for Health Sciences, Riyadh, Saudi Arabia; 2 King Abdullah International Medical Research Center, Riyadh, Saudi Arabia; 3 Ministry of the National Guard Health Affairs, Riyadh, Saudi Arabia; 4 Restorative and Prosthetic Dental Sciences Department, College of Dentistry, King Saud bin Abdulaziz University for Health Sciences, Riyadh, Saudi Arabia

**Keywords:** artificial intelligence, assessment, automated, dental students, ergonomics, evaluation, musculoskeletal disorders, work postures

## Abstract

**Background:**

Dental professionals are frequently exposed to work-related musculoskeletal disorders (WMSDs), with ergonomic risks often emerging during undergraduate dental training. While most existing studies rely on self-reported questionnaires, objective evaluations of work postures remain limited. This study aimed to evaluate work postures adopted by dental students while performing restorative procedures in simulation laboratories using an AI-based assessment model.

**Methods:**

A cross-sectional analytical study was conducted among dental students. Participants were recruited through a non-probability purposive sampling method. They were photographed while performing dental procedures in a simulation laboratory. The images were analyzed using a previously validated AI-based Assessment Model [SBK-DentErgo].

**Results:**

A total of 75 students were included in the final analysis. Regarding overall postural aspects, it is noteworthy that none of the students demonstrated acceptable posture. The mean score for the overall posture analysis was 14.2 (±1.6). Among the postural aspects, the hip was highly compromised in 55 (73.3%), followed by upper arm in 52 (64.3%), wrist 48 (64%), neck (side to side) 36 (58.7%), elbows 38 (50.7%), neck (front) 36 (48%). Fisher's Exact test showed D3 students significantly had acceptable hip angulation posture, 10 (52.6%) acceptable, compared to D2 students, among whom 16 (88.9%) exhibited compromised hip angulation (*p* = 0.02). Similarly, 12 (63.2%) D3 students demonstrated an acceptable wrist position, whereas D4 students, 16 (88.9%), had a compromised wrist position (*p* = 0.01). Gender-based postural assessment revealed a significant difference in wrist positioning: male students, 19 (52.8%), maintained acceptable wrist position, compared to 31 (79.5%) female students who exhibited compromised wrist angulation (*p* = 0.01). Multinomial regression analysis indicated that D3 students had higher odds of maintaining appropriate hip (OR = 6.29, *p* = 0.01, 95% CI: 1.37–28.85) and wrist (OR = 6.85, *p* < 0.01, 95% CI: 1.62–28.89) postures, while female students demonstrated lower odds of maintaining appropriate wrist posture.

**Conclusions:**

Dental students adopt improper postures during dental procedures, indicating an early risk of developing ergonomic problems during their training. This AI-model is effective for objective evaluation of ergonomic work postures and can serve as supplementary educational tool for detecting postural deviations in educational settings.

## Introduction

Dentistry requires operating in a confined, hard-to-access oral cavity, often forcing clinicians into static, asymmetrical, and improper postures (1). These unsuitable positions are a leading contributor to musculoskeletal pain in the profession (2, 3). Sustained and repeated stress on the neck, shoulders, trunk, and waist exacerbates work-related musculoskeletal disorders (WMSDs), highlighting a significant occupational health burden (4–6). Common resulting conditions include tension neck syndrome, muscle stiffness, and hand–wrist disorders such as tendinitis, tenosynovitis, De Quervain's disease, trigger finger, carpal tunnel syndrome, and Guyon's syndrome (7).

Numerous studies have highlighted the widespread prevalence of musculoskeletal disorders (MSDs) among dental students worldwide. A study conducted in Australia reported that 85% of dental students experienced MSDs (8). Similarly, research in Brazil found that 82.6% of dental students suffered from MSDs (9). In the USA, the prevalence was 61% among dental students (10). In Iran, it was 82% (11), and in India, 71.1% (12). Furthermore, 90.2% of dental students in the United Arab Emirates (UAE) were reported to suffer from MSDs (13). Additionally, research by AlSahiem J. et al. found that the prevalence of MSDs among dental students in Saudi Arabia was 87% (14). MSDs significantly affect dental students, causing pain, reduced quality of life, negative impacts on academic performance, increased sick leave, and absenteeism. These issues may ultimately influence their future careers, potentially leading to early retirement or disability (15). Several other studies have also indicated that these MSDs frequently begin during university education (3, 4, 8, 16). It has also been reported that although the risks associated with MSDs are communicated to and understood by students in the early phases of their training, this occupational health issue tends to receive diminishing attention over time (17).

Considering the detrimental effects of incorrect work postures on the health and careers of students, it is crucial to evaluate the postures adopted by dental students throughout their training. This assessment can help identify and correct improper postural habits before they result in permanent injuries (18, 19). Nevertheless, most studies documented in the literature rely on questionnaires that assess self-reported musculoskeletal disorders (MSDs) among dental students (2, 4–6, 8, 14, 20). Only a limited number of studies have attempted to analyze the work postures of dental students using photographic assessments or direct observation; however, these methods are often subject to observer bias and inter-examiner variability (21–26).

Recent technological advancements in healthcare, particularly the application of artificial intelligence (AI) in dentistry, have demonstrated exceptional performance in diagnosing oral diseases, treatment planning, and predicting disease prognosis. These studies have indicated that AI models can serve as adjunctive tools to assist healthcare providers (27, 28). To address the limitations of the human observations in postural assessment, we recently developed an AI-based deep learning model to evaluate the ergonomic work postures of dental professionals. The model was previously validated and demonstrated high sensitivity (97%) and specificity (85.7%) in classifying ergonomic postures, supporting its use as an adjunctive assessment tool (29).

Considering the performance of the AI model, our study aimed to assess the work postures of dental students while performing restorative procedures in simulation laboratories using this AI-based assessment model.

## Materials and methods

### Research design and setting

A cross-sectional analytical study was conducted in accordance with the Declaration of Helsinki. Prior to the commencement of the research, ethical approval was obtained from the Institutional Review Board of the King Abdullah International Medical Research Center, Riyadh, Saudi Arabia (KAIMRC) (IRB No. NRR24/053/12, approved on 22 December 2024). This study was carried out in the preclinical simulation laboratory of the College of Dentistry at King Saud bin Abdulaziz University for Health Sciences in Riyadh, Saudi Arabia.

### Sample size estimation

The sample size was determined based on ANOVA (Fixed effects, omnibus, one-way) with study power of 80% and a 95% confidence interval and aneffect size of 0.40, the sample size was selected based on a previous study assessing ergonomic posture outcomes among dental students (26). Sample size estimation was performed using G*Power software (version 3.1.9.4). The required sample size for this study was 78 dental students.

### Sampling technique

The students were recruited from four different academic levels: second-, third-, and fourth-year dental students, as well as interns. A non-probability purposive sampling method was employed. All eligible students from each academic level were invited to participate, and those who consented were included until the desired sample size was reached, with a minimum of 20 students recruited from each academic level.

### Eligibility criteria

Inclusion Criteria: Students who were willing to participate and signed the written informed consent form, and who have passed the preclinical operative dentistry course in the first year (D1) of the dental program.

Exclusion Criteria: Students who were unwilling to be photographed, with a history of diagnosed MSDs and those undergoing treatment for MDSs.

### Data collection tool

Data were obtained using a personal information form that included participants' demographic details along with a written informed consent form.

### Data collection

The eligible participants were photographed while performing dental procedures on phantom heads in the preclinical simulation laboratories. The procedural task for the students was standardized; that is, the students were instructed to prepare Class I amalgam conservative cavity preparations on the right lower first molar (typodont tooth #46) mounted on the Planmeca Compact Dental Simulator (Planmeca Dental Products, Helsinki, Finland). A total of 15 min was allocated for this procedure. Photographs were taken simultaneously, 5 min after the student began the procedure. The students were unaware of the timing of the photograph capture.

Photographs were taken using two iPhone 15 devices (Apple Inc., Cupertino, CA, USA). Both devices were mounted on a leveled tripod: one positioned on the left side (sagittal plane) at a 90-degree angle to capture the side profile, and the other facing the operator (frontal plane) to record the front view. The cameras were positioned 1.5 meters from the dental chair at a height of 1 meter to capture the participants' working area, thereby facilitating the collection of data required for the study. These methods were selected based on a review of previous studies (25, 29–31). No intrinsic or extrinsic camera calibration was performed because the AI pipeline relies on relative joint angles derived from 2D pose estimation rather than absolute spatial measurements. This approach aligns with prior ergonomic assessment studies and the previously validated version of the model (29).

All photographic data were managed in accordance with institutional ethical guidelines to ensure confidentiality. Images were captured to minimize identifiability by focusing on body posture and avoiding facial features whenever possible. Photographs were neither blurred nor digitally altered, as such modifications would have compromised the accuracy of AI-based landmark detection and posture evaluation. Prior to analysis, images were anonymized using unique study codes without any personal identifiers and stored on password-protected devices and secure institutional servers accessible only to the research team. The images were used exclusively for approved research purposes.

The photographic records were subsequently uploaded for evaluation using the Dental Ergonomic Posture Assessment Model [SBK-DentErgo], which was developed by effectively integrating YOLOv11 (You Only Look Once Version 11) and MediaPipe to ensure precise and reliable human pose estimation. This system calculates angles between anatomically significant joint triplets through vector-based geometric analysis. Each computed angle is classified into ergonomic risk zones—acceptable, compromised, or harmful—based on recognized ergonomic standards adapted from the Modified Dental Operator Posture Assessment Criteria (M-DOPAC) (29). Ideal postures are illustrated in both lateral and frontal views. Each element is assigned a score within one of three categories: acceptable (1 point), compromised (2 points), or harmful (3 points). Only six components are categorized as harmful. Consequently, scores range from 10 to 26, with the most optimal postures earning a score of 10 points. Scores between 11 and 20 are considered compromised, while scores of 21 and above are identified as harmful. Consequently, a participant could demonstrate one or more acceptable postural components while still being categorized as having an overall compromised posture (Table 1).

**Table 1:** Modified-dental operator posture assessment criteria (M-DOPAC) (29).

Posture apects	Scoring criteria		
	Acceptable (1 Point)	Compromised (2 Points)	Harmful (3 Point)
HIPS	Level on stool	Hips not level on stool	Not applicable
TRUNK	Front to back ≤20°	Front to back >20° < 45°	Front to back ≥45°
	Side to side ≤20°	Side to side >20° < 45°	Side to side ≥45°
HEAD/NECK	Front to back ≤20°	Front to back >20° < 45°	Front to back ≥45°
	Side to side ≤20°	Side to side >20° < 45°	Side to side ≥45°
SHOULDERS	Relaxed	Slummed forward	Not applicable
	Both shoulders level with trunk	One or both shoulders elevated above line of trunk	Not applicable
UPPER ARMS	Upper arms parallel to long axis of torso	<20° abduction away from body	>20° abduction awayfrom body
	Elbows at waist level	Elbows at waist level but <60°	Elbows at waist level but >60°
WRIST	Flexion or extension of either wrist ≤15°	Flexion or extension of either wrist >15°	Not applicable

**Figure 1:**
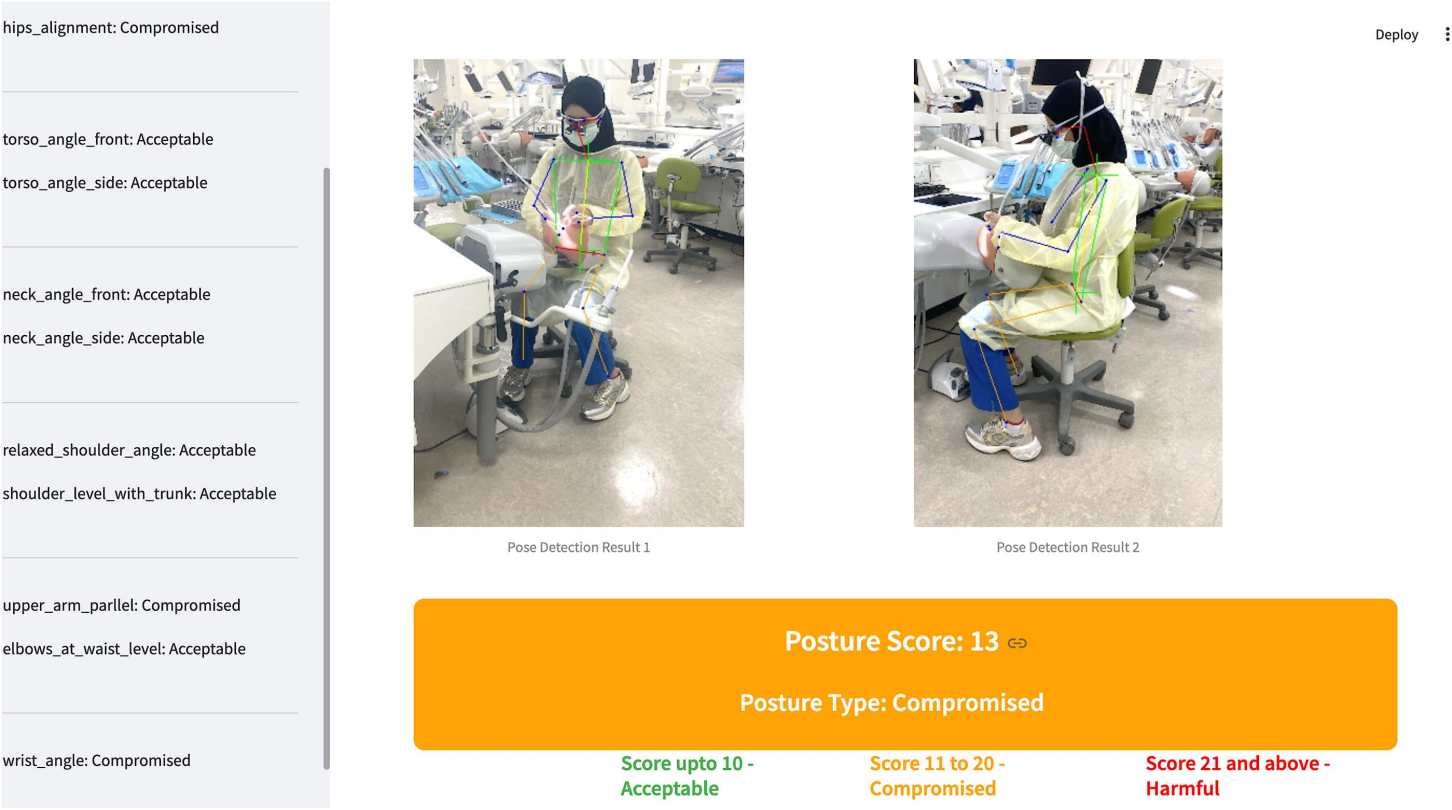
Evaluation of ergonomic work postures using an AI-based based assessment model [SBK-DentErgo].

The scores obtained from the model were entered into a Microsoft Excel sheet (Figure 1). Occlusion and missing keypoints were addressed using MediaPipe's internal confidence scoring system. Images in which one or more critical landmarks necessary for posture classification could not be reliably detected due to occlusion or segmentation failure were excluded from the analysis. As the model had been previously validated with high sensitivity (97%) and specificity (85.7%) (29), no retraining or recalibration was performed for the present dataset. In the present study, 82 students agreed to participate; however, seven participants were excluded from the analysis because one ergonomic parameter—upper arms parallel to the long axis of the torso—could not be detected by the AI model. This occurred when the arms were positioned too close to the torso, causing visual occlusion and limiting segmentation. Therefore, only the evaluation scores of 75 participants were subjected to analysis.

### Data analysis

The data collected were transferred into SPSS Statistical Software version 29 (IBM Corporation, Armonk, NY, USA). Data cleaning was conducted prior to the transfer for analysis. Descriptive statistics were recorded. Mann–Whitney “*U*” test and Fisher's Exact test were used to assess the associations between postural aspects and independent variables. The ergonomic posture score served as the dependent variable, while gender and academic level were considered independent variables. Multinomial logistic regression was conducted to evaluate the odds ratios of significant independent variables influencing ergonomic posture scores.

## Results

The present study included 75 students, of whom 39 (52%) were male and 36 (48%) were female. The academic representation was nearly equal across each academic year: D2 had 18 (24%), D3 had 19 (25.3%), D4 had 18 (24.0%) and interns also had 20 (26.7%) students. Nine of the ten postural aspects recorded during cavity preparation—hip, torso (front to back), neck (front), shoulder (relaxed), shoulder (level with the trunk), upper arm, elbows, and wrist—were either compromised or harmful, except for the torso when assessed side to side, which was observed in all 75 (100%) students. Among the postural aspects, the hip was highly compromised in 55 (73.3%) students, followed by the upper arm 52 (64.3%), wrist 48 (64%), neck (side to side) 36 (58.7%), elbows 38 (50.7%), neck (front) 36 (48%), shoulder (relaxed) 2 (2.7%), shoulder (level with the trunk) 1 (1.3%), and torso 1 (1.3%) students. The neck posture, when assessed from the front in 16 (21.3%) students and from the side in 6 (8.0%), was the most associated with harmful posture adoption (Table 2).

**Table 2:** Individual demographic variables and posture aspects description.

Variable	Total (N)		75	Frequency (n)		Percentage (%)
Gender	Male		75	39		52.0
	Female			36		48.0
Academic Level	D2		75	18		24.0
	D3			19		25.3
	D4			18		24.0
	Internship			20		26.7
Hip	Acceptable		75	20		26.7
	Compromised			55		73.3
Torso front to back	Acceptable		75	74		98.7
	Compromised			01		1.3
Torso side to side	Acceptable		75	75		100
Neck Front	Acceptable		75	23		30.7
	Compromised			36		48.0
	Harmful			16		21.3
Neck Side	Acceptable		75	25		33.3
	Compromised			44		58.7
	Harmful			06		8.0
Shoulder angle (relaxed)	Acceptable		75	73		97.3
	Compromised			2		2.7
Shoulder angle (level with trunk)	Acceptable		75	74		98.7
	Compromised			1		1.3
Upper Arm	Acceptable		75	22		29.3
	Compromised			52		69.3
Elbows	Acceptable		75	37		49.3
	Compromised			38		50.7
Wrist	Acceptable		75	27		36.0
	Compromised			48		64.0
Overall Analysis (Total of Posture Aspects)	Compromised		75	75		100.0
	Minimum	Maximum		Mean	(±S.D)	
Overall Posture Aspects	11	18		14.2	(1.6)	
Mann Whitney “*U*” test	No Loupes	With Loupes	75	Mean Rank		*P* Value
	70	5		38.30	33.80	0.65

When considering overall postural aspects, none of the students demonstrated either acceptable or completely harmful posture. Instead, all 75 students (100%) exhibited compromised posture. The mean score for the overall posture analysis was 14.2 (±1.6). Although certain individual postural components were classified as acceptable, the cumulative ergonomic scores for all participants fell within the compromised range. Consequently, no participant met the criteria for either a fully acceptable or a fully harmful overall posture classification. Among these students, five used head-mounted loupes while preparing cavities. We assessed whether there was a significant difference in posture between those who used loupes and those who did not. The Mann–Whitney *U* test indicated a lower mean rank for students using loupes (33.80) compared to those without loupes (38.30), although this difference was not statistically significant (*p* = 0.65). This lack of significance may be due to the small sample size (Table 2).

Fisher's Exact test was used to assess the association between postural aspects and independent variables (educational level and gender). D3 students were significantly more likely (*p* = 0.02) to have an acceptable hip angulation posture, with 10 (52.6%) classified as acceptable, compared to D2 students, among whom 16 (88.9%) exhibited compromised hip angulation. Similarly, a significant number of D3 students 12 (63.2%) demonstrated an acceptable wrist position (*p* = 0.01), whereas the majority of D4 students 16 (88.9%) had a compromised wrist position (Table 3).

**Table 3:** Fishers exact test for association of posture with independent variables.

Educational level (variable)	Posture aspects	Acceptable	Compromised	Total	df	Fishers exact test *P*-value
D2	Hip	2 (11.1)	16 (88.9)	18 (100)	3	0.02*
D3		10 (52.6)	9 (47.4)	19 (100)		
D4		5 (27.8)	13 (72.2)	18 (100)		
Interns		3 (15)	17 (85)	20 (100)		
Total		20 (26.7)	55 (73.3)	75 (100)		
D2	Wrist	9 (50)	9 (50)	18 (100)	3	0.01*
D3		12 (63.2)	7 (36.8)	19 (100)		
D4		2 (11.1)	16 (88.9)	18 (100)		
Interns		4 (20)	16 (80)	20 (100)		
Total		27 (36)	48 (64)	75 (100)		

Gender		Acceptable	Compromised	Harmful	Total	df	Fishers exact test *P*-value
Female	Upper Arm	8 (20.5)	31 (79.5)	0 (0)	39 (100)	2	0.08
Male		14 (38.9)	21 (58.3)	1 (2.8)	36 (100)		
Total		22 (29.3)	52 (69.3)	1 (1.3)	75 (100)		
Female	Wrist	8 (20.5)	31 (79.5)	—	39 (100)	1	0.01*
Male		19 (52.8)	17 (47.2)	—	36 (100)		
Total		27 (36.0)	48 (64.0)	-	75 (100)		

* *P*-value<0.05.

A significant gender-based difference was observed in wrist positioning (*p* = 0.01). Male students were more likely to maintain an acceptable wrist position during cavity preparation, with 19 (52.8%) demonstrating acceptable positioning, compared to female students, among whom only 8 (20.5%) exhibited acceptable wrist posture. Conversely, 31 (79.5%) female students demonstrated compromised wrist posture, compared to 17 (47.2%) male students (Table 3).

A multinomial regression analysis was conducted to evaluate the association between the significant independent variables identified by Fisher's exact test (academic year and gender) and postural aspects, comparing acceptable posture with compromised postural adaptation. D3 students were more likely to maintain appropriate hip (OR: 6.29, *p* = 0.01, 95% CI: 1.37–28.85) and wrist (OR: 6.85, *p* < 0.01, 95% CI: 1.62–28.89) positions during cavity preparation compared to students from other academic years. Gender comparison revealed that females had lower odds of maintaining the appropriate wrist position during cavity preparation (OR: 0.23, *p* < 0.01, 95% CI: 0.08–0.63) (Table 4).

**Table 4:** Multinomial regression analysis of significant independent variables with posture assessment criteria.

Independent variable		Posture aspect	Position assessment based on criteria	Exp(B)	df	Sig.	Exp(B)	95% confidence interval for Exp(B)	
								Lower bound	Upper bound
academic year	D2	HIP	Acceptable	0.70	1	0.72	0.70	0.10	4.80
	D3			6.29	1	0.01*	6.29	1.37	28.85
	D4			2.17	1	0.34	2.17	0.43	10.83
	Interns			0^a^	—	—	—	—	—
Academic Year	D2	Wrist	Acceptable	4.00	1	0.58	4.00	0.95	16.76
	D3			6.85	1	0.00*	6.85	1.62	28.89
	D4			0.50	1	0.45	0.50	0.08	3.12
	Interns			0^a^	—	—	—	—	—
Gender	Female	Wrist	Acceptable	0.23	1	0.00	0.23	0.08	0.63
	Male			0^a^	—	—	—	—	—

B, odds.

* *P*-value<0.05.

a Reference category: compromised.

## Discussion

Dental students undergo an intensive phase of professional development that involves rigorous training and clinical practice, placing significant strain on the musculoskeletal system. Prolonged dental procedures performed in suboptimal postures make students particularly susceptible to musculoskeletal pain, commonly affecting the neck, shoulders, and wrists. Early identification of improper postural habits and the integration of ergonomic training at the undergraduate level are essential to promote healthy work postures and prevent the development of work-related musculoskeletal disorders throughout their professional careers.

This study evaluated the ergonomic work postures of dental students during simulated dental procedures using an AI-based posture assessment model [SBK-DentErgo]. The findings indicate that a substantial proportion of students adopted non-ergonomic postures. The results are concerning, as none of the students demonstrated an overall acceptable posture. Based on the predefined scoring thresholds, all participants were classified within the compromised overall posture category. However, although the cumulative posture scores fell within the compromised range, certain individual postural components—particularly the neck and wrist—were observed to be within the harmful category in a subset of students.

Among the postural aspects, the hip was the most compromised in students, followed by the upper arm, wrist, and neck. Neck posture was the factor most strongly associated with the adoption of harmful postures. D3 students were significantly more likely to maintain an acceptable work posture compared to D2 and D4 students. Gender-based postural assessment revealed a significant difference in wrist positioning between male and female students. Male students were more likely to maintain an acceptable wrist position while preparing the cavity compared to female students.

These findings are consistent with those reported by Araújo, M.S. et al., who evaluated work postures using photographs and found that more than half of the dental students in Brazil exhibited incorrect postures (22). Our results also align with those reported by Corrales Zúniga, I.A. et al., who assessed work postures through photographs and found that over half of the dental students in Managua, Central America, adopted harmful postures (25). Similar findings were reported by Manchi-Zuloeta et al., with more than 70% of dental students in Lima exhibiting harmful work postures (32).

In the present study, we observed that D3 students were significantly more likely to maintain an acceptable work posture compared to D2 and D4 students. This difference may be attributed to the inclusion of an Ergonomics course at the D3 level in the curriculum. However, it is noteworthy that D4 students did not maintain an acceptable work posture as well as D3 students. Contrary to expectations that ergonomic awareness improves with training, the findings suggest that posture-related risks persist across all academic levels. Our results also align with those reported by Corrales Zúniga, I.A., et al., who found that over half of the fifth-year students exhibited highly compromised ergonomic work postures compared to fourth-year students. Therefore, although the risks associated with MSDs are taught and understood by students during the early stages of their training, occupational health concerns tend to receive less attention over time (33).

In the present study, gender comparison revealed that females had lower odds of maintaining the appropriate wrist position during cavity preparation compared to male students. This could be one of the factors contributing to the higher incidence of MSDs reported among female students compared to males, as noted by Almeida, M.B. de, who found that female students had a higher prevalence of MSD symptoms than their male counterparts (34). Another study by Alsulaihebi, H.S. et al. also reported that female students exhibited significantly higher MSD scores compared to their male peers (35). Previous ergonomic research indicates that hand anthropometric characteristics and strength differ significantly between males and females, with males exhibiting greater hand strength than females. This difference may influence tool handling and wrist postures during fine motor tasks, such as those performed in dentistry. These factors support the notion that the observed gender differences in wrist posture may be influenced not only by individual operator technique but also by operator anthropometry (36).

Given the widespread occurrence of compromised work postures in the present study, it is important to recognize that improper postural deviations are directly linked to specific biomechanical stressors. These deviations alter the normal distribution of forces, resulting in muscle imbalances, increased joint stress, and the potential for microtrauma to tissues (37). Therefore, if these issues are not identified and addressed early, they can become significant risk factors for the development of MSDs (37). The early assessment of improper work postures and the implementation of scientifically grounded corrective measures have become critical for reducing the incidence of MSDs (38, 39). However, it is important to note that MSDs in dental students are multifactorial and cannot be attributed solely to clinical posture. Prolonged static postures during studying and other daily activities may also contribute to cumulative musculoskeletal strain. In addition to posture correction, preventive strategies should include individualized musculoskeletal strengthening and conditioning exercises tailored to the individual, which may help improve physical resilience and reduce strain.

In earlier research, most data were collected through self-reported questionnaires, visual observations, or photographic evaluations, all of which are susceptible to observer bias and variability among examiners (21–24). In contrast, the current study employed an AI-driven assessment model, enabling an objective and consistent evaluation of work postures, thereby minimizing observer bias and enhancing standardization. The implementation of AI-based posture assessment could serve as a valuable supplement to ergonomic education by providing objective feedback to students throughout their preclinical training.

In the present study the seven participants were excluded because the AI model was unable to detect the upper arm position when the arms were too close to the torso. In future studies, this limitation may be addressed by incorporating additional camera angles and depth-sensing technologies to reduce occlusion-related detection errors. Expanding the AI training dataset to include occluded upper limb postures could further enhance detection accuracy. These methodological refinements would improve the robustness and applicability of AI-based ergonomic assessments in dental education settings.

This study encountered several limitations that should be considered when interpreting the results. First, a cross-sectional design was employed, which limits the ability to establish causal relationships between ergonomic work postures and potential musculoskeletal outcomes. Second, the evaluation was conducted in a preclinical simulation laboratory using phantom heads, which may not fully capture the complexity, time constraints, and ergonomic demands of real clinical settings, thereby limiting the generalizability of the findings. Third, although an AI-based posture assessment model was used to enhance objectivity, the system relied on two-dimensional image capture and was susceptible to detection errors caused by occlusion. Consequently, a small number of participants were excluded because the model failed to identify upper arm posture when the arms were positioned close to the torso. This exclusion was methodological rather than behavioral, resulting from visual occlusion when the upper arms were positioned very close to the torso. Consequently, these participants could not be accurately scored using the current AI Model. Although this exclusion reduced the final sample size, it is unlikely to have introduced significant selection bias, as the excluded cases were not systematically associated with specific gender or academic characteristics. However, it is possible that students adopting more constrained or ergonomically suboptimal arm positions were disproportionately affected, which could lead to a slight underestimation of the prevalence of compromised upper arm posture. Consequently, the findings should be interpreted with caution when generalizing to postures involving arm proximity to the torso. Additionally, the study was performed at a single institution, which may restrict its external validity. Lastly there could be potential Hawthorne effect despite partial nondisclosure. Furthermore, ergonomic considerations extend beyond individual posture to include workstation design, seating, instrument ergonomics, and lighting conditions, all of which may interact with posture and influence musculoskeletal stress.

Future studies should employ longitudinal designs and validate AI-driven ergonomic assessments in real clinical settings to improve generalizability. Methodological enhancements, such as incorporating multi-angle or 3D pose estimation techniques, are recommended to reduce detection errors caused by occlusion. Future studies could benefit from using multi-camera systems or depth-based sensors to enhance landmark detection and minimize data exclusion. Expanding the size of training datasets and correlating AI-generated posture scores with musculoskeletal symptoms will further clarify their clinical relevance. Additionally, randomised controlled trials evaluating AI-assisted ergonomic feedback interventions during dental training are essential.

### Educational implications and implementation plan

The results of this study highlight the potential benefits of incorporating AI-driven ergonomic posture assessments into undergraduate dental education. Since poor posture is common at all academic levels, it is important to introduce early and structured ergonomic interventions. AI tools such as SBK-DentErgo can be integrated into preclinical training through a gradual, formative process. In the initial phases, AI-based posture analysis can be integrated into ergonomics courses to enhance students’ understanding of proper working postures by providing personalized visual feedback after simulated procedures. During intermediate training, regular AI assessments can be incorporated into routine simulation lab sessions to monitor posture over time and reinforce ergonomic principles. At advanced stages, AI-generated posture data can be combined with faculty support to deliver targeted ergonomic training and workstation adjustments. Crucially, AI-based assessments should complement, rather than replace, traditional teaching methods by providing objective, standardized feedback that encourages lasting ergonomic habits and may help reduce the risk of musculoskeletal disorders over time. While the present study highlights the potential of AI-assisted posture assessment, the long-term effectiveness of such interventions has yet to be established. Future longitudinal studies are necessary to determine whether improvements in posture are sustained over time.

## Conclusions

This research revealed that a significant number of dental students exhibited compromised postures. Notably, none of the students demonstrated acceptable work postures while performing simulated dental procedures, highlighting the presence of ergonomic risk postures during these simulations. The implementation of this AI–based posture assessment model has proven effective for the objective evaluation of ergonomic work postures. Furthermore, the results support the viability of AI-assisted assessment as a supplementary educational tool for detecting postural deviations in educational settings.

## Data Availability

The original contributions presented in the study are included in the article/Supplementary Material, further inquiries can be directed to the corresponding author.
